# Insect Vectors of Disease: Untapped Reservoirs for New Antimicrobials?

**DOI:** 10.3389/fmicb.2016.02085

**Published:** 2016-12-22

**Authors:** Nicholas J. Tobias

**Affiliations:** Molekulare Biotechnologie, Fachbereich Biowissenschaften, Goethe-Universität FrankfurtFrankfurt am Main, Germany

**Keywords:** natural products, symbiosis, microbiome, secondary metabolites, non-ribosomal peptide synthetase, polyketide synthase

## Abstract

With the increase in antibiotic resistance among infectious diseases, the need for new strategies for identifying compounds with inhibitory effects is dire. Traditional methods of genome sequencing and systematic characterization of potential antimicrobial gene clusters, although effective, are unfortunately not yielding results at a speed consistent with the rise in antimicrobial resistance. One approach could be to use a more targeted approach to antimicrobial compound discovery. Insect vectors often mediate the transmissions of parasitic infections for example, leishmaniasis, malaria, and trypanosomiasis. Within the insect, among the pathogens, are commensal and/or obligate symbiotic bacteria, which often undergo a population shift upon colonization of the insect host. The remaining bacteria may be either resistant to the effects of the respective parasites, or possibly essential for a successful colonization and continued spread of the parasite due to an obligate symbiosis between bacteria and insect host. Interestingly, these shifts are often toward groups of bacteria known to harbor many polyketide synthase and non-ribosomal peptide synthetase gene clusters involved in bioactive molecule production. This perspective explores the possibility to exploit the natural interactions between parasite, symbiont, and insect host for a more targeted approach toward natural product discovery, in an environment where potential compound producers are naturally interfacing with human pathogens.

## Introduction

Antibiotic resistance is estimated to cause approximately 10 million deaths per year by 2050 (O’Neill, 2016). Despite this, natural compounds with antibiotic properties are widespread in the environment, produced by organisms found in environments ranging from deep-sea water in the Arctic (Yuan et al., 2014) to plants in tropical rainforests (Qin et al., 2009). However, the major impediment to new antimicrobials in clinical use, is that the discovery of new compounds with potential clinical applications is simply too slow to keep up with the rise of resistance. Natural product research has now developed into an enormous field and is significantly simplified with the advent of easily accessible, low-cost, high-throughput sequencing technologies. This simplification would suggest that we should be finding novel antibiotics much faster than previously, which unfortunately is not currently the case.

Insects, like other eukaryotes, have a lifestyle and diet that is often complemented (and influenced) through interactions with microorganisms (Yun et al., 2014). Whether supplying enzymatic capabilities, nutrient exchange, defense against competing organisms, or host signaling, mutualistic symbioses are widespread in nature. Underlying these interactions are complex layers of regulation in both the host and their normal flora. However, the gut-microbial population is in a constant state of flux and the composition can be heavily influenced by diet (Muegge et al., 2011).

Insects are the responsible vectors for several parasitic diseases including African sleeping sickness (African trypanosomiasis), Chagas disease (American trypanosomiasis), malaria, and leishmaniasis. The responsible insects are very different for each disease, maintain different feeding patterns and therefore have their own unique repertoire of microbial flora. However, what they all have in common is a blood feeding stage in their life cycle. Following a blood meal, the microbial contents of the gut changes (Wang et al., 2011; Diaz-Albiter et al., 2012; Buarque et al., 2016) and in some cases, this microbial shift may affect the outcome of successful colonization by parasites (Tchioffo et al., 2013; Geiger et al., 2015). The mechanism(s) of support for the host are not always fully elucidated. Candidates for involvement in these processes are secondary metabolites (SMs), which are generally considered to be non-essential to survival. However, these natural products, often produced by either non-ribosomal peptide synthetases (NRPSs) or polyketide synthases (PKSs), have a well described role in supporting symbioses between vertebrates and either bacteria or fungi (Beemelmanns et al., 2016). NRPSs and PKSs are large protein complexes, which produce small compounds that often have medically relevant biological activities. Here, the idea that some of these SM may contain activity against invading parasites within the gut of different insect species is explored.

## Microbiome of *Lutzomyia longipalpis*

*Lutzomyia longipalpis* is a major vector responsible for transmission of visceral Leishmaniasis. Early on, studies in phlebotomines related to *L. longipalpis*, highlighted an interesting observation regarding the microflora of *Phlebotomus* females – where large amounts of bacteria were present in the gut, *Leishmania* transmission was impacted in a negative way (Schlein et al., 1985). What followed after many years, was a study in *L. longipalpis*, where bacterial symbionts were found to defend directly against colonization of *Leishmania*. The authors noted that where bacteria were present prior to exposure of the sandfly to *Leishmania, Leishmania* infection was less likely to establish a foothold within the gut of the sandfly (Sant’Anna et al., 2014). This observation is not surprising, nor is it an isolated incident with increased microbial diversity in a locust gut found to also be protective against invasion of pathogens (Dillon et al., 2005). Conversely, and perhaps more interestingly, when the roles were switched and *Leishmania* first colonized the sand fly gut, the parasite provided protection against bacterial pathogens (Sant’Anna et al., 2014). The finding that microbial flora can be protective leads to the obvious question of how? A study taking inventory of the species present by mapping shotgun transcriptome data to the nucleotide databases provided some insights (McCarthy et al., 2011). Although not a targeted approach specifically for symbiont detection, the study identified several bacteria and fungi that contain SM gene clusters. Presumably, the bacteria identified are those present in higher numbers due to the global approach (insect transcripts accounted for the majority of reads) the researchers took. However, examining available genomes of those bacteria and fungi named, account for 70 SM clusters, collectively (**Table 1**) with potentially interesting bioactivities. With high bacterial loads being somewhat protective against parasitic colonization followed by the fact that the bacterial load contains a reasonably large number of SM producing gene clusters, it seems reasonable to expect that at least some of these bacteria may produce metabolites that somehow, specifically inhibit the parasite.

**Table 1:** Break down of secondary metabolite (SM) clusters identified from bacteria and fungi highlighted in metagenomic studies focusing on insect vectors of Leishmaniasis and Chagas disease.

Insect species	Insect flora	NRPS	PKS	Bacteriocin	Other	Total	Reference
*Lutzomyia longipalpis*	*Anoxybacillus flavithermus*	0	1	2	3	6	NC_011567.1
	*Ralstonia pickettii*	0	1	0	3	4	NC_010682.1
	*Propionibacterium acnes*	1	0	1	1	3	NC_006085.1
	*Geobacillus kaustophilus*	0	1	2	1	4	NC_006510.1
	*Streptomyces coelicolor*	3	8	2	14	27	NC_003888.3
	*Leifsonia xyli*	0	0	0	2	2	NC_022438.1
	*Acinetobacter baumannii*	2	0	0	3	5	NZ_CP009257.1
	*Cunninghamella bertholletiae*	1	0	0	5	6	JNEG00000000.1
	*Mortierella verticillata*	4	0	0	9	13	AEVJ00000000.1
**Total**		**11**	**11**	**7**	**41**	**70**	McCarthy et al., 2011
*Triatoma* spp.	*Corynebacterium stationis*	0	1	0	2	3	CP014279.1
	*Corynebacterium casei*	0	1	0	3	4	NZ_CP004350.1
	*Corynebacterium glutamicum*	0	1	0	3	4	NC_022040.1
	*Gordonia* sp. KTR9	7	1	1	6	15	NC_018581.1
	*Dietzia maris*	1	1	0	5	7	NZ_LVFF00000000.1
	*Serratia marcescens* WW4	4	0	0	0	4	NC_020211.1
**Total**		**12**	**5**	**1**	**19**	**37**	Gumiel et al., 2015


*Representative species mentioned in analysis of the visceral Leishmaniasis vector, *Lutzomyia longipalpis* (McCarthy et al., 2011) and two vectors for Chagas disease, *Triatoma brasiliensis* and *Triatoma pseudomaculata* (Gumiel et al., 2015) were used in antiSMASH v3.0 (Weber et al., 2015) to identify secondary metabolite clusters. Where a specific strain was not named, a representative genome from the species was used as an example. All references refer to GenBank accession numbers. The ‘other’ column includes all those clusters not falling into one of NRPS, PKS or bacteriocin.*

## Microbiome of *Anopheles*

Studies frequently focus on the microbial diversity of insect species, with their specific roles not definitively determined. One exhaustive study examined the microbiome of *Anopheles* and nicely analyzed much of the data available, identifying a total of 109 unique genera with many different reported effects on the fitness of *Plasmodium* spp. but no overall role for the symbionts (Villegas et al., 2014). Although we should be best placed to determining the role of symbionts in mosquitos given the extensive number of studies performed, the complexity of the microbiome in different species has complicated this. Despite different mosquito species occupying very different niches and developing under different conditions, Proteobacteria appear to be the most frequently identified phylum (Minard et al., 2013). Within the Proteobacteria, there do exist examples of anti-parasite producing flora strengthening the idea that these environments could be a rich source of new anti-parasitic compounds. One example is *Serratia marcescens* that was isolated from *Anopheles albimanus* and found to be capable of eviscerating the oocyst development of *Plasmodium vivax* (Gonzalez-Ceron et al., 2003) as well as showing some anti-trypanosomal activity in the gut of *Rhodnius prolixus*, a common Chagas disease insect vector (Azambuja et al., 2004). *S. marcescens* is commonly found in the environment and is a known SM producer. One possibility explaining the production of anti-parasitic compounds under certain environmental conditions is niche adaptation in some strains, thereby allowing the bacteria a selective advantage in colonizing the gut of these insects.

## Microbiome in Chagas Disease Vectors

*Trypanosoma cruzi* is the causative agent of Chagas disease and is spread by various species of triatomine. *Triatoma infestans* and *R. prolixus* are two common triatomine species that spread the disease in South and Central America, respectively. Survival of these insect vectors of Chagas disease are known to be dependent on their bacterial symbionts, at least for the production of B-complex vitamins that are typically lacking from the blood diet maintained by the insects (Baines, 1956; Harington, 1960). In *R. prolixus*, the bacteria determined to be responsible for this is the actinomycete, *Rhodococcus rhodnii* (Baines, 1956). Interestingly, these bacteria are not obligate symbionts of the Chagas disease vectors, *T. infestans* (*Nocardia* sp.), *Panstrongylus megistus* (*Gordonia* sp.) or *Triatoma sordida* (*Rhodococcus equi*) (Eichler and Schaub, 2002). Members of the genera associated with these other symbionts are, however, known prolific SM producers. The question as to whether or not this is simply a coincidence is a valid one. One cannot, however, ignore the fact that the effect on these symbionts upon ingestion of a given parasite results in an initial decrease in symbiont numbers, followed by recovery and subsequently significantly higher numbers of symbiotic bacteria, suggesting that these symbionts are somewhat resilient to parasitic attack (Eichler and Schaub, 2002). The decrease in microbe numbers has been attributed to the triggering of antimicrobial peptides by the incoming parasite. However, not all bacteria are affected and prior to the recovery reported by Eichler and Schaub (2002), pyrosequencing of the *R. prolixus* gut compartments demonstrates a decrease in the Enterococcaceae and Mycobacteriaceae populations but an increase in Burkholderiaceae members and no change in the Nocardiaceae or Enterobacteriaceae in *Trypanosoma rangeli* infected insects, resulting in a net increase of SM producing bacterial families (Vieira et al., 2015). The role of SM in this process is as yet unknown, however, there are many SM playing roles in defense against various competitors in similar interactions in the environment (Beemelmanns et al., 2016).

The observation of SM-producing insect microbiota is continued beyond just the symbionts of the triatomines. A metagenomic study exploring other gut microbiota of *Triatoma* spp. collected from peridomestic habitats highlighted an over-abundance of actinomycetes and Proteobacteria (Gumiel et al., 2015). The potential SM clusters from the bacteria identified (whether transient or commensal) in this study, totaled 37 (**Table 1**). In fact, upon the uptake of *T. cruzi* by insects, the gut flora has shown to be, in some instances, naturally protective against *T. cruzi.* This was shown to be through the production and secretion of prodigiosin, a SM produced by *S. marcescens* (Azambuja et al., 2004). In addition to the SM producing bacteria, an earlier study analyzing the mycobiome of five separate *Triatoma* spp. identified a total of 393 individual fungal strains predominantly from the genera *Penicillium, Aspergillus*, and *Acremonium* (Moraes et al., 2001), which are all known to be prolific producers of SM, further supporting the antimicrobial potential of these insect flora.

## Future Perspectives and Concluding Remarks

It is well known that gut microbiota play important roles in protecting against disease (Azambuja et al., 2004; Yilmaz et al., 2014). This is not limited, however, to just insect guts, but is indeed also true in the human gut (Shreiner et al., 2015). The maintenance of SM gene clusters, coupled with the proven ability of microbial flora to protect against diseased states raises the question as to the specific role of SM in these symbioses. Why are organisms with such a number of SM gene clusters consistently maintained while others are lost? Perhaps this is entirely a coincidence, however, coupled with the important role of SM in other symbioses (Beemelmanns et al., 2016), this point is worth further investigation.

Products of NRPSs and PKSs have been touted as one of the major hopes for new antibiotic discovery in the current generation. Indeed many current antibiotics are products (or derivatives) of these enzymatic complexes. Despite this, resistance to multiple current antibiotics used for specific disease is continually increasing, for example tuberculosis (Migliori et al., 2007) and gonorrhea (Unemo and Nicholas, 2012). Next generation sequencing has generated a huge influx of data and created a major bottleneck in product discovery from these NRPS or PKS clusters, particularly those that have potential for use in human medicine. One interesting, albeit underrepresented approach in the literature, is a functionally targeted approach to natural product discovery where investigations of microbial sources are performed in the context of natural interactions between symbiont and human pathogen. With a population shift upon infection of parasites in insects, one might ask the question, why do some bacteria survive while others appear to be completely removed from the environment? It is known that parasites produce antimicrobial compounds that are probably responsible for this shift, either directly or indirectly (Castro et al., 2012; Vieira et al., 2015). However, there is also the possibility that in fact, there is a complex signaling pathway in place that leads to the removal of specific non-essential flora. It makes sense that these compounds produced by the parasite are somehow targeting bacteria that would otherwise impede its entrance and replication within the insect. Yet how are these bacteria being depleted? Is it a direct response from the parasite? Or is it an indirect response caused by the presence of the insect on other commensal bacteria causing them to produce compounds such as bacteriocins?

Secondary metabolites from some bacteria are already known to influence insect immune systems (Downer et al., 1997; Crawford et al., 2012; Dudnik et al., 2013). This theme of SM maintenance may thus prove to simply be essential for optimizing conditions for parasite colonization. Regardless, understanding the complex interactions that occur in microbial-invertebrate interactions could be one important step to understanding both how and why these populations are shifting. The mechanisms which make these particular species apparently immune from removal following parasite entry is one which should be explored as it may additionally provide leads as to how to prevent the human stage of parasite replication, or at least shift the balance in favor of clearance from the host. This understanding could also be exploited by equipping commensal organisms with the ability to stimulate insect immune responses in a non-beneficial way toward the incoming parasite to prevent parasite colonization, or offensive tools that directly target the parasites as they are acquired in a paratransgenic approach. Indeed, some studies have already identified anti-parasitic compounds, as discussed above, yet the full potential of the microbiome and particularly the mycobiome has yet to be exploited.

## Author Contributions

The author confirms being the sole contributor of this work and approved it for publication.

## Conflict of Interest Statement

The author declares that the research was conducted in the absence of any commercial or financial relationships that could be construed as a potential conflict of interest.
